# Inhibition of Zbp1-PANoptosome-mediated PANoptosis effectively attenuates acute pancreatitis

**DOI:** 10.1038/s41420-025-02451-7

**Published:** 2025-04-16

**Authors:** Jie Li, Yu-chen Jia, Jiongdi Lu, Haoyu Zhang, Zheng Wang, Xiaozhou Xie, Feng Cao, Fei Li

**Affiliations:** 1 Department of General Surgery, Xuanwu Hospital, Capital Medical University, Beijing, China; 2 Clinical Center for Acute Pancreatitis, Capital Medical University, Beijing, China

**Keywords:** Cell death, Acute pancreatitis

## Abstract

Early acute pancreatitis is an acute inflammatory disease that involves multiple modes of cell death, including apoptosis, necrotic apoptosis, and pyroptosis in its disease process. PANoptosis, a type of cell death that includes pyroptosis, apoptosis, and necroptosis, has had an important role in a variety of infectious and inflammatory diseases in recent years. To judge the relationship between PANoptosis and AP, we first analyzed the data from pancreatic transcriptome data by bioinformatics techniques, and we found the enrichment of PANoptosis pathway in AP. Next, we screened the genes and identified differentially expressed genes (DEGs) associated with AP and PANoptosis. Finally, we found that Zbp1 may have a major role in the process of PANoptosis. For this purpose, we constructed AP models in mice and in vitro cell line 266-6 and intervened by inhibiting Zbp1. The final results showed that the PANoptosis in mice was significantly suppressed after inhibition of Zbp1. In conclusion, inflammatory injury in AP can be significantly improved by inhibiting Zbp1- PANoptosome-mediated PANoptosis.

## Introduction

Acute pancreatitis (AP) is a common acute abdominal disease, the incidence of which is increasing year by year, and the prognosis of patients with severe disease is poor [1, 2]. The pathological features of AP are edema and necrosis of acinar cells, as well as inflammatory cell infiltration of pancreatic tissue and hemorrhage, and so on. In particular, the death of acinar cells is an important factor that exacerbates the process of AP [3]. Damaged or dead acinar cells release damage-associated pattern molecules (DAMPs), which are recognized by specific pattern recognition receptors (PRRs) in the host and exacerbate the inflammatory response, leading to the release of pro-inflammatory factors that trigger and exacerbate the inflammatory response, causing further damage to pancreatic tissue [4–6]. Recent studies have shown that there are multiple modes of programmed cell death in the process of acinar cells, such as necrosis, apoptosis, pyroptosis, ferroptosis, and necroptosis, among others [3, 7–10]. The many programmed deaths synergistically induce acinar cell death toward death and promote inflammatory progression.

PANoptosis is a unique regulatory cell death modality that does not a single mode of death but emphasizes the concept of apoptosis, necroptosis, and pyroptosis as a synergistic cell death modality [11, 12]. PANoptosis combines the key features of pyroptosis, apoptosis, and necroptosis and has been implicated in the development of a wide range of human diseases, such as infections, cancers, inflammatory injuries, etc. PANoptosis involves the assembly of a class of complex multiprotein complexes (PANoptosome) that are essential for initiating cell death and sensing DAMPs or other risk factors [13–16]. It has now been shown that the PANoptosome can contain molecules such as Ripk1, Ripk3, ASC, Nlrp3, Casp1, Casp8, Casp6, Zbp1, etc., and that the formation of the PANoptosome activates pyroptosis, apoptosis, and necroptosis, which cause programmed cell death [17–19]. Zbp1 plays an important role, and its role as a receptor for Z-DNA or Z-DNA can effectively activate inflammation [20, 21]. However, in recent years, it has been shown that Zbp1 can also recognize Z-type nucleic acids (Z-NA) in the nucleus, triggering the activation of its inflammatory pathway, which is a new extension for the study of some inflammatory diseases [22]. Our study suggests that the formation of Zbp1-PANoptosome will have an important effect on PANoptosis during AP development and can promote acinar cell injury.

## Results

### Establishment of mouse AP model and enrichment of PANoptosis pathway

**Fig. 1:**
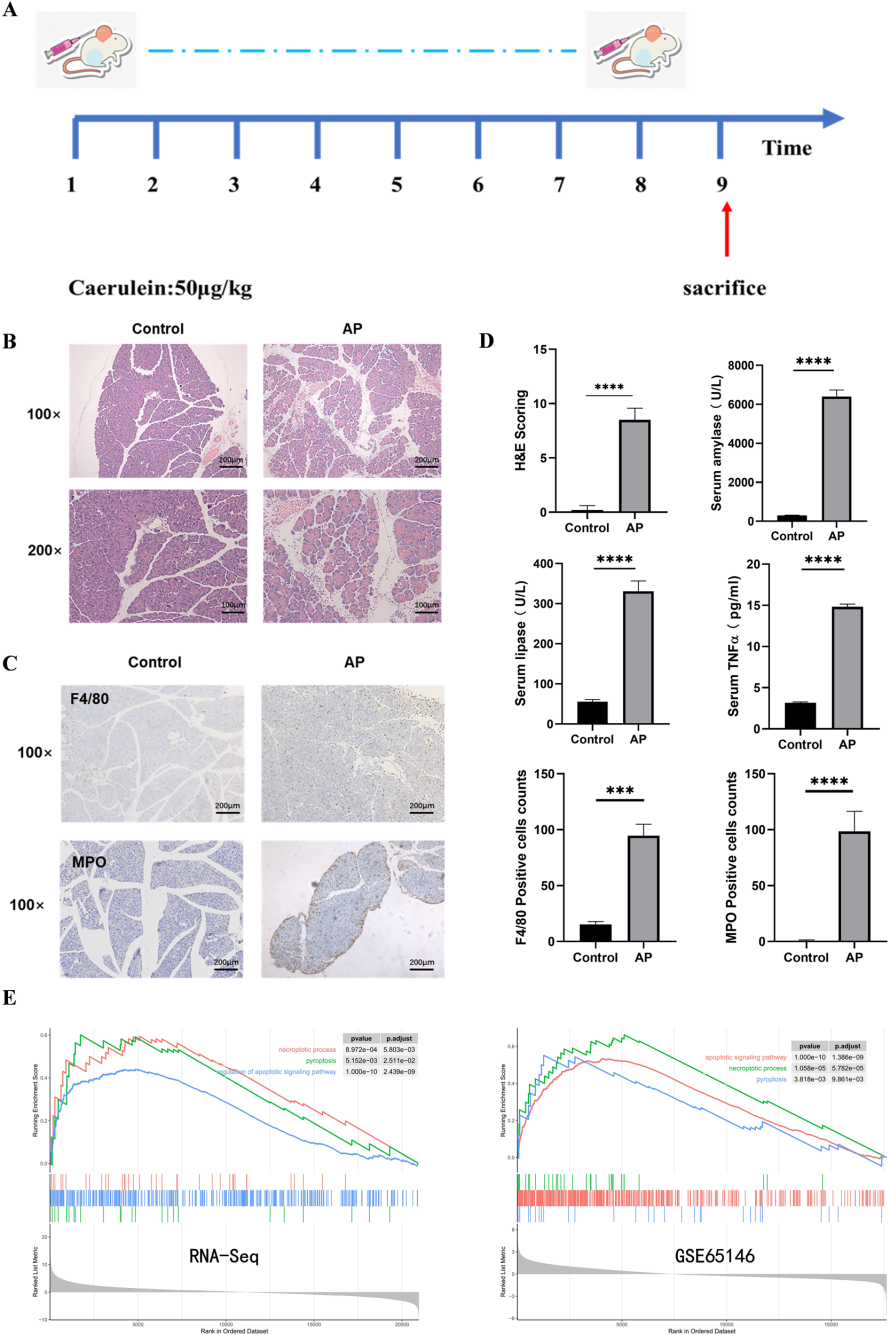
Establishment of mouse AP model and enrichment of PANoptosis pathway. **A** Construction of the mouse AP model. **B** H&E staining of mouse pancreatic tissue. **C** IHC staining of macrophage and neutrophil. **D** Pathological scores of staining and expression levels of serum amylase, lipase, and TNFα in mice. **E** GSEA enrichment analysis of transcriptome sequencing data and GSE65146 data for PANoptosis in mouse pancreas.

We established an AP model in mice by 8 injections of caerulein (50 μg/kg) and killed the mice at the 9th hour to obtain serum and pancreatic tissues (Fig. 1A). First, pathological staining of pancreatic tissues and IHC of macrophages showed that the tissues were edematous, infiltrated with inflammatory cells, and had a small amount of necrosis and hemorrhage during AP. Serum amylase and lipase were elevated in the AP group, and the inflammatory factor TNFα was also significantly elevated (Fig. 1B–D). Using the constructed model and the control group, we performed transcriptome sequencing. Analysis of the data revealed that PANoptosis was enriched in AP, and pathways exhibiting apoptosis, necroptosis, and pyroptosis were significantly enriched and elevated. To validate our data results, we simultaneously analyzed mouse AP data using the GSE65146 database and found the same PANoptosis enrichment at AP. This result illustrates the importance of PANoptosis as a mode of cell death in AP (Fig. 1E).

### Gene screening of PANoptosis and identification of key gene Zbp1

**Fig. 2:**
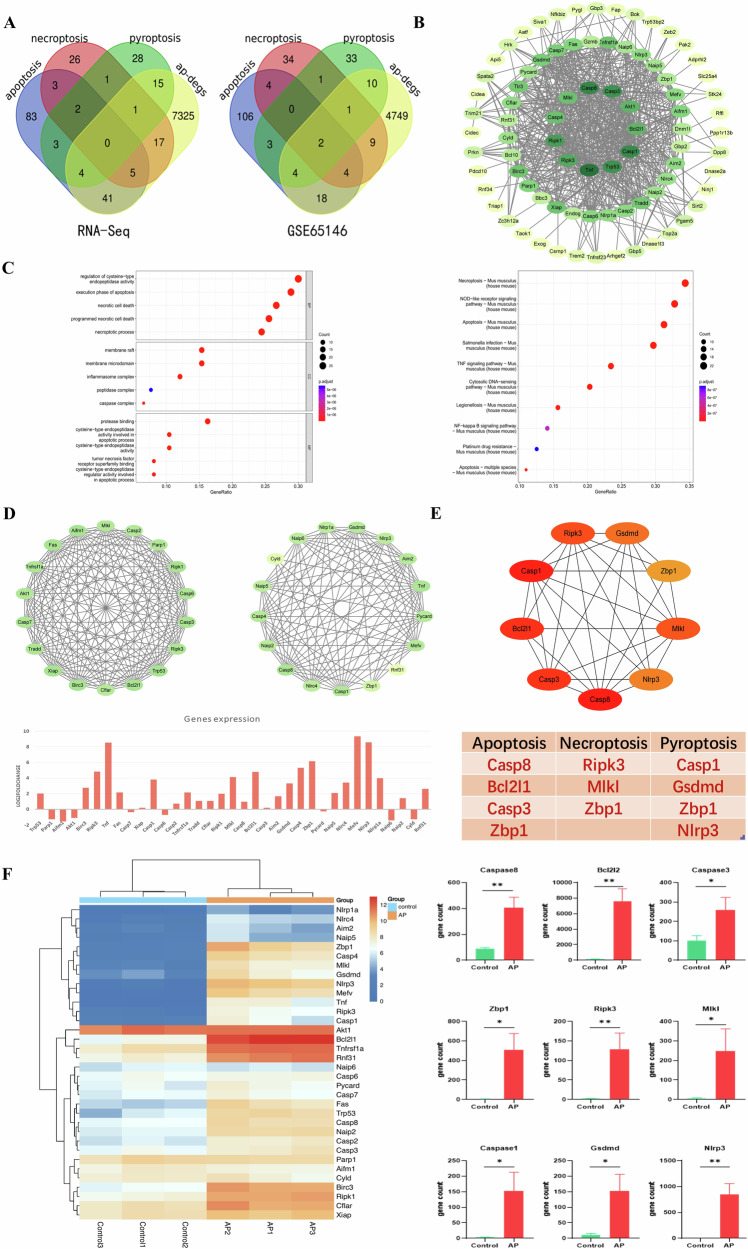
Gene screening of PANoptosis and identification of key gene Zbp1. **A** Venn diagram of transcriptome sequencing data and GSE65146 data of mouse pancreas with PANoptosis gene set. **B** Protein Interaction Networks for DEGs. **C** Map of GO and KEGG pathways for DEGs. **D**, **E** Hub genes obtained by Cytoscape software analysis. **F** Heatmap of core genes and histogram analysis of focused genes.

To further investigate the specific role of PANoptosis in AP, we performed further analysis. DEGs obtained from the comparison of AP and control groups were intersected with genes of PANoptosis, and a total of 108 DEGs were obtained (Fig. 2A, B). GO and KEGG enrichment of these differential genes revealed that the key pathways were focused on cell death (Fig. 2C). To further analyze the key genes, we performed protein interaction analysis using STRING database, and after further screening by MCODE function of cytoscape software, we obtained 35 key genes. We analyzed and finally selected 9 key genes (Casp8, Ripk3, Casp1, Bcl2l2, Casp3, Mlkl, Gsdmd, Zbp1, Nlrp3) for validation (Fig. 2D, E). Our transcriptomic data showed that these 9 key genes were all upregulated at the time of AP, demonstrating a PANoptosis-like cell death that is significantly expressed at the time of AP. We also found that Zbp1 was simultaneously involved in all three cell death modes, apoptosis, necrotic apoptosis, and pyroptosis, and may be involved in the disease process of AP as a central regulatory gene of PANoptosis (Fig. 2F).

### Protein expression of key genes for PANoptosis

**Fig. 3:**
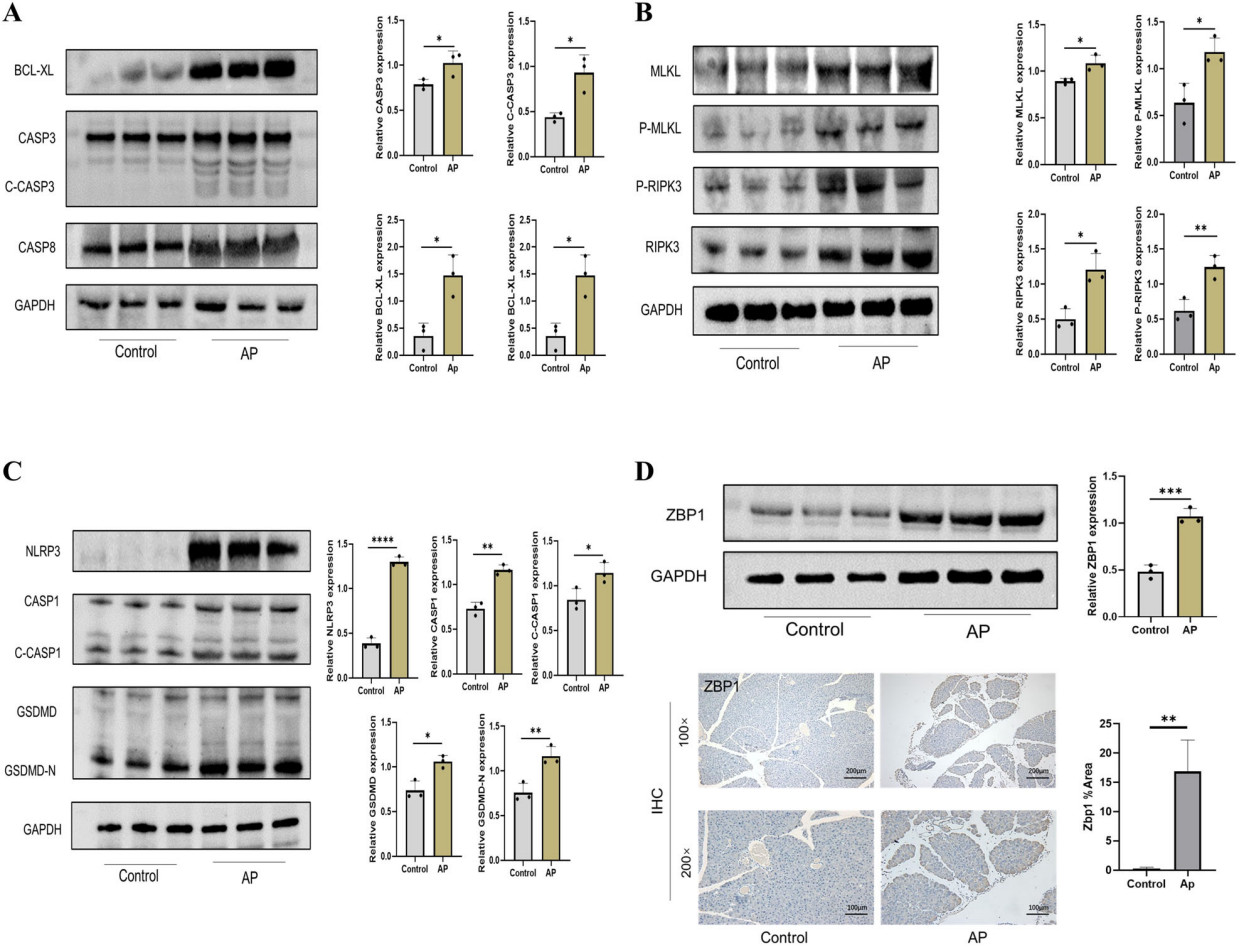
Protein expression of key genes for PANoptosis. **A** Expression of apoptosis-related proteins in normal and AP groups of mouse pancreas. **B** Expression of necroptosis-related proteins in normal and AP groups of mouse pancreas. **C** Expression of pyroptosis-related proteins in normal and AP groups of mouse pancreas. **D** Protein expression and IHC staining of Zbp1.

To further substantiate our results, we examined pancreatic tissue from a mouse AP model by Western blot. Of the nine genes screened above, we examined apoptosis (Bcl-2, Casp3, Casp8), necrotic apoptosis (Mlkl, Ripk3), and pyroptosis (Nlrp3, Casp1, Gsdmd) separately. The results show that the expression of apoptosis-related proteins Bcl2l2, Casp3, Casp8, and cleaved Casp3 were significantly increased in AP (Fig. 3A); the expression of key proteins of necroptosis, Mlkl, Ripk3, and their phosphorylated proteins, P-Mlkl and P-Ripk3, were significantly upregulated in AP (Fig. 3B); and the expression of key proteins of pyroptosis, Nlrp3, Casp1, Gsdmd as well as cleaved Casp1 and cleaved Gsdmd (Gsdmd-N) all showed significantly elevated status in AP (Fig. 3C). We also verified the status of our key protein Zbp1, which was significantly upregulated in AP as shown by protein and IHC results (Fig. 3D). In summary, the results showed that apoptosis, necroptosis and pyroptosis proteins were significantly elevated in AP pancreatic tissues at the time of AP, suggesting that PANoptosis-like cell death is more prevalent at the time of AP.

### Inhibition of Zbp1 attenuates the inflammatory state and PANoptosis during AP in mice

**Fig. 4:**
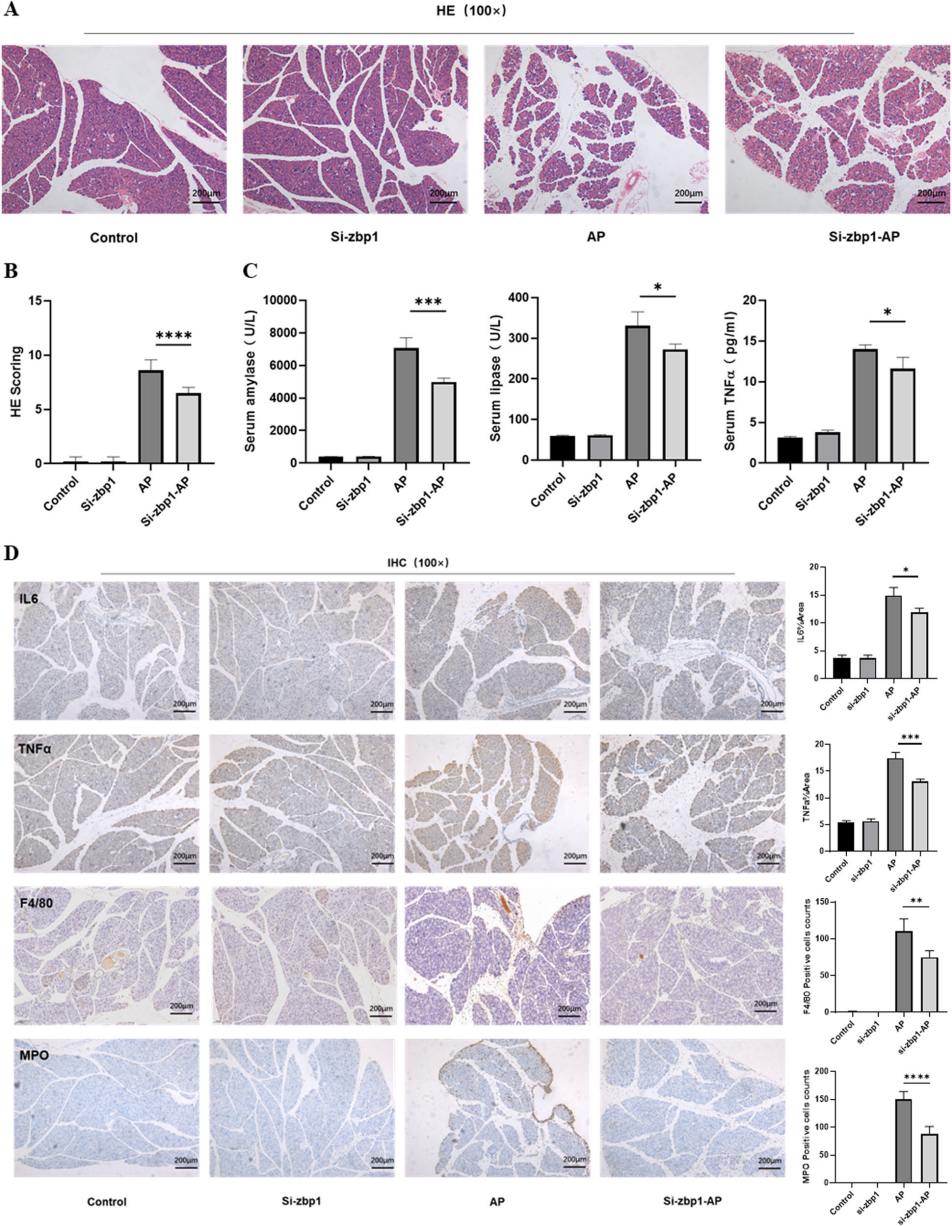
Inhibition of Zbp1 attenuates the inflammatory state in mice. **A** H&E staining of the mouse pancreas. **B** Pathologic scoring of the mouse pancreas. **C** Serum amylase, lipase and TNFα expression levels in mice. **D** IHC staining of mouse pancreatic tissue for TNFα, IL6, F4/80 and MPO.

**Fig. 5:**
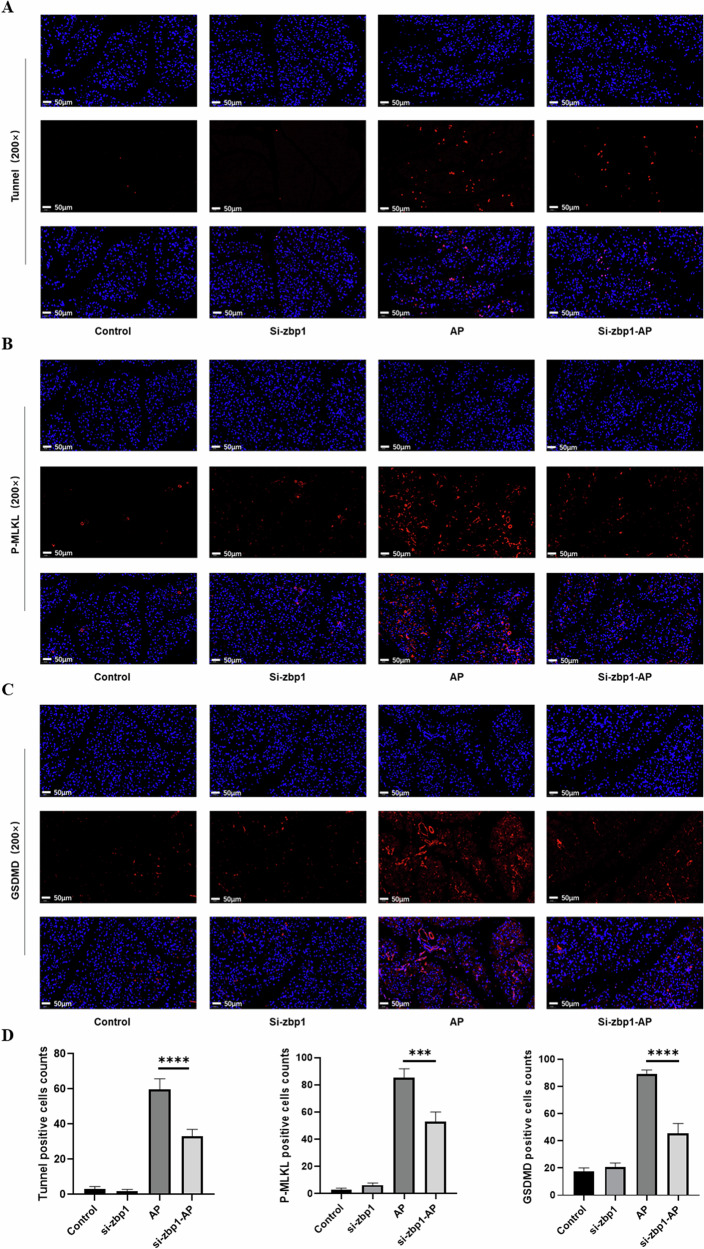
Inhibition of Zbp1 attenuates PANoptosis in mice. **A** Tunnel staining of mouse pancreas. **B** Fluorescence staining of P-mlkl, a key gene for necroptosis. **C** Fluorescence staining of the pyroptosis-related gene Gsdmd. **D** Statistical scoring of immunofluorescence.

To verify whether Zbp1 is a critical gene in PANoptosis-like cell death in AP, we intervened in mice. We found that after reducing the expression of Zbp1, the degree of inflammation in mice with AP was somewhat reduced (Fig. 4A, B). The pathological score of H&E was reduced in the si-Zbp1-AP group compared with the AP group, while the expression of serum amylase, lipase, and the inflammatory factor TNFα were improved (Fig. 4C); IHC of pancreatic tissues also showed that the level of inflammation, as well as the degree of inflammatory cell infiltration in the pancreas, were reduced to some extent (Fig. 4D). To assess the occurrence of PANoptosis-like cell death, we performed immunofluorescence detection experiments for three key indicators, specifically tunnel staining to detect apoptosis, P-Mlkl to detect necrotic apoptosis, and finally Gsdmd was used to observe pyroptosis (Fig. 5A–C). The results showed that apoptosis, necroptosis and pyroptosis of pancreatic acinar cells were significantly improved after inhibition of Zbp1 (Fig. 5D). These results demonstrated that Zbp1, as a central gene, plays an important role in activating PANoptosis in acinar cells.

### Zbp1-PANoptosome can activate Nlrp3 to promote inflammation

**Fig. 6:**
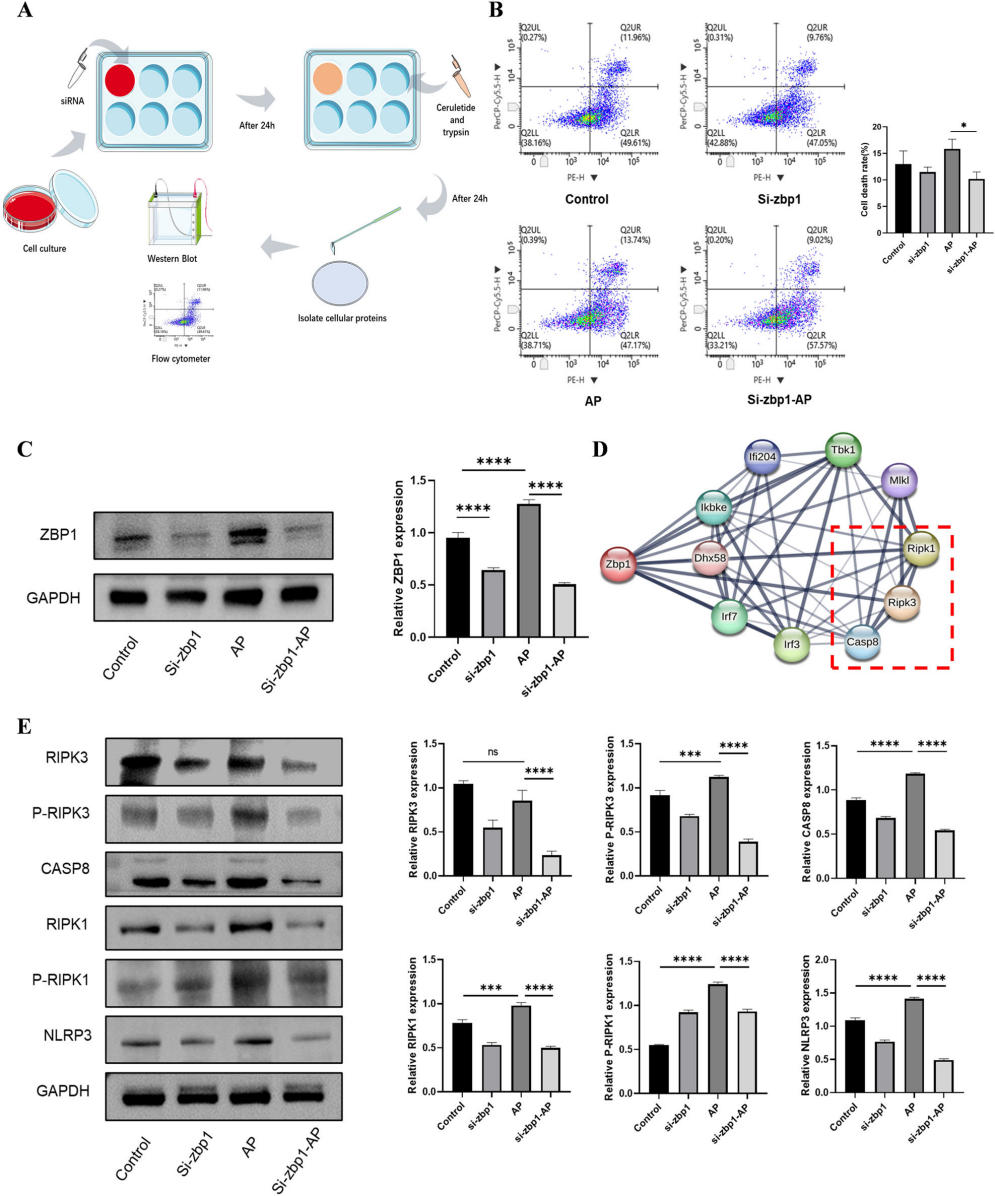
Zbp1-PANoptosome can activate Nlrp3 to promote inflammation. **A** Model construction and experimental procedure for 266-6 cells. **B** The level of cell death after siRNA transfection in each group. **C** Protein expression levels of Zbp1 in each group after siRNA interference. **D** The protein interaction network of Zbp1. **E** Expression levels of PANoptosis-related proteins in each group after siRNA transfection.

We performed mechanistic studies using the 266-6 cell line to observe changes in the PANoptosis pathway upon inhibition of Zbp1. We performed gene silencing of Zbp1 using siRNA and then constructed AP models (Fig. 6A, C). Flow cytometry was used to examine apoptosis and necrosis of the cells. The results showed that the rate of late apoptosis and necrosis (Q2UR) was significantly reduced after Zbp1 inhibition compared to the AP group (Fig. 6B). We analyzed the gene correlations of Zbp1 using STRING database, and we found that three genes, Ripk1, Ripk3 and Casp8, were more associated with Zbp1 (Fig. 6D). We then silenced the gene expression using siRNA, and the effect was remarkable. Next, we performed protein assays on Ripk1, Ripk3, and Casp8, and the results showed that after knocking down the expression of Zbp1, the expression of Ripk1, Ripk3, and Casp8, as well as that of P-Ripk1 and P-Ripk3, showed a significant decrease (Fig. 6E). This suggests that after inhibition of Zbp1, it may down-regulate PANoptosis-like death via the Ripk1-Ripk3-Casp8 complex. Meanwhile, the expression of Nlrp3 was significantly reduced after inhibition of Zbp1, and the results suggest that the Zbp1-Ripk1-Ripk3-Casp8 complex is involved in Nlrp3 inflammatory vesicle-dependent acinar cell death (Fig. 6E).

**Fig. 7:**
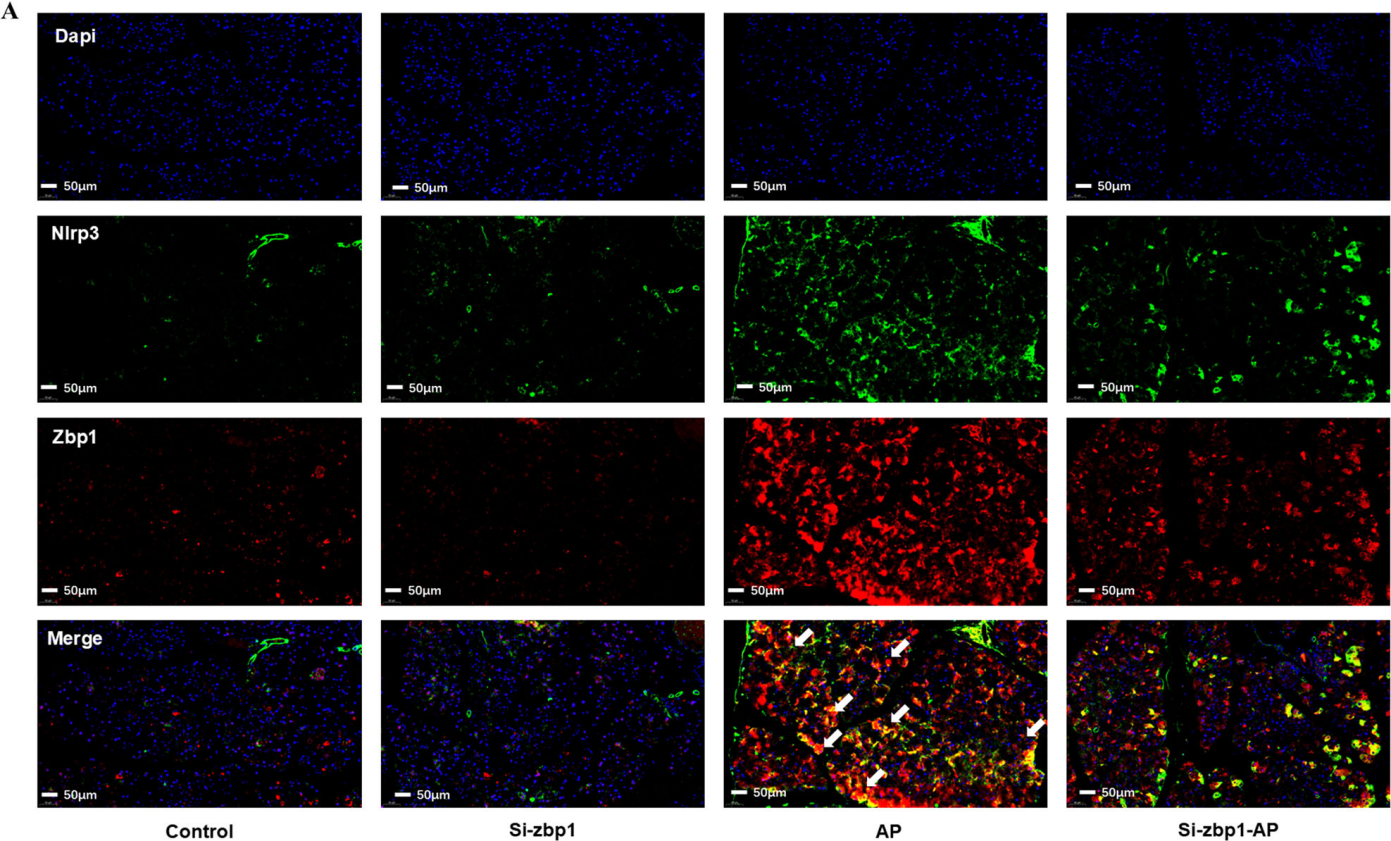
Relationship between Zbp1 and Nlrp3. **A** Immunofluorescence co-localization of Zbp1 and Nlrp3 in various subgroups. Green fluorescence is for Nlrp3, and red fluorescence is for Zbp1.

In addition, to further clarify the relationship between Zbp1 and Nlrp3, we did immunofluorescence co-localization in mouse pancreatic tissues. The results showed that there was a large amount of co-localization between Zbp1 and Nlrp3 during the process of AP (Fig. 7A). This result fully illustrated that Zbp1-triggered PANoptosis might occur through Nlrp3 inflammatory vesicles.

## Discussion

AP is a common abdominal surgical emergency, and its current incidence is about (4.9–73.4)/100,000, with an increasing trend year by year. As the disease progresses, about 20% of patients will develop symptoms such as pancreatic and peripancreatic tissue necrosis or organ failure, with a mortality rate of 20–40% [2]. Approximately 30% of these patients will have secondary infection and develop infected pancreatic necrosis, with a mortality rate of up to 30% [23]. Therefore, the diagnosis and treatment of AP remains a major challenge. Currently, acinar cell death is an important factor in the development of acute pancreatitis, and its specific mechanism is not well understood. Recent studies have shown that there are multiple modes of cell death during the course of AP, such as apoptosis, necroptosis, pyroptosis, and ferroptosis. Thus, a single mode of death cannot exist in isolation and there must be a common mechanism. A new programmed cellular death approach that combines apoptosis, necroptosis, and pyroptosis as modes of cell death is called PANoptosis [19, 24, 25]. PANoptosis requires the involvement of the PANoptosome, which provides a molecular scaffold for key molecules involved in pyroptosis, apoptosis, and necroptosis simultaneously. These include Nlrp3, Casp1, and Gsdmd (pyroptosis), Casp8, Casp3, and Casp7 (apoptosis), and Casp8, Ripk1, Ripk3, and Mlkl (necroptosis) [26, 27]. Together, these molecules form the PANoptosome protein complex and further contribute to the activation of downstream cell death effector molecules that enhance the inflammatory response [21, 28, 29]. Our study shows that PANoptosis plays an important role in the developmental progression of AP, where apoptosis, necroptosis, and pyroptosis coexist. We have shown that proteins comprising the PANoptosome are simultaneously activated in AP, including Bcl-XL, Casp8, and Casp3 (apoptosis), Ripk3 and Mlkl (necroptosis), and Nlrp3, Casp1, and Gsdmd (pyroptosis). Together, the activation of these proteins promotes the inflammatory response in AP.

**Fig. 8:**
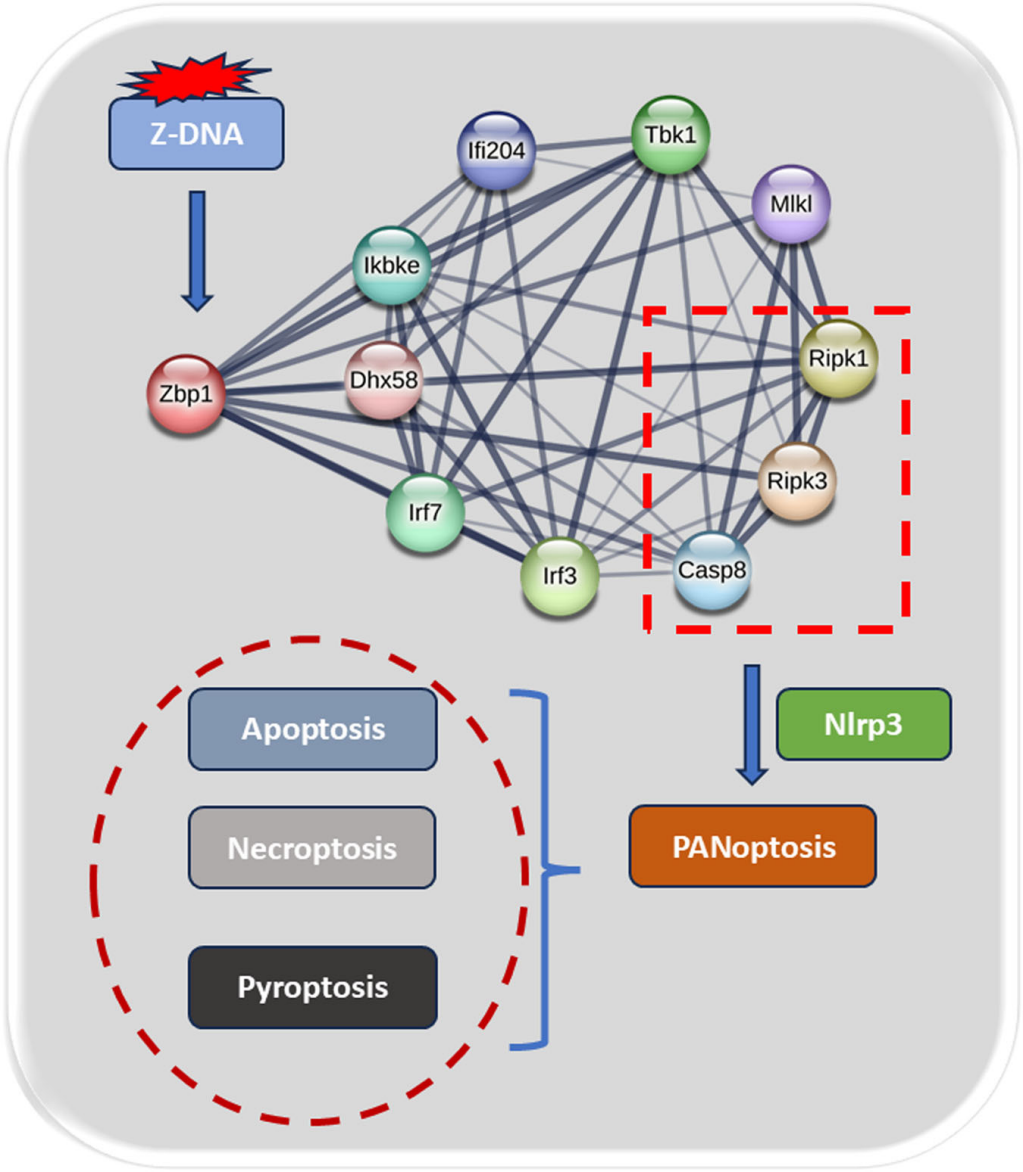
Diagram of the mechanism by which ZBP1 regulates PANoptosis in AP.

Zbp1 is an innate immune receptor that senses nucleic acids and activates PANoptosis, and its activation leads to the recruitment of Ripk3 and Casp8, which interact with their receptors to form a scaffold for cell death signaling [30–33]. The Zbp1-PANoptosome complex is involved in Nlrp3 inflammatory vesicle-dependent focal death, Casp8-mediated apoptosis, and Ripk3/Mlkl-driven necroptosis [20, 26, 34]. In our study, we identified a central role of Zbp1 in the AP process of PANoptosis through a detailed analysis of transcriptomic genes. Its ability to sense nucleic acids released by cellular damage during inflammation initiates the assembly of the PANoptosome, which in turn promotes the death of acinar cells, thereby amplifying the inflammatory response. Upon inhibition of Zbp1, we observed a reduction in inflammatory injury in the mouse pancreas, with some improvement in the degree of edema, necrosis, and inflammatory cell infiltration in the pancreatic tissue. In addition, serum amylase lipase and inflammatory factor TNFα were reduced to varying degrees. This suggests an important role for Zbp1 in initiating and promoting inflammatory pathways. To observe the downstream function of Zbp1, we performed fluorescence detection of apoptosis, necroptosis, and pyroptosis in pancreatic tissues. The results showed that inhibition of Zbp1 significantly reduced PANoptosis in pancreatic acinar cells (Fig. 8).

The death pathway mediated by Zbp1-PANoptosome is well known to be involved in NLRP3 inflammatory vesicle-dependent cell death [34, 35]. We gained insight into Zbp1 from the STRING database and found that it is closely related to Ripk1, Ripk3, and Ripk8. We used the mouse acinar cell line 266-6 for molecular studies by transfecting siRNAs to interfere with the gene. We found that the expression of Ripk1, Ripk3, Ripk8, and Nlrp3 were all significantly reduced after the inhibition of Zbp1 expression compared with the AP group. Flow cytometry showed that the number of cell necrosis was also slightly reduced in the siRNA group. Therefore, the inflammatory pathway mediated by Zbp1-PANoptosome plays an important role in the development of PANoptosis, and inhibition of this pathway may play an important role in the improvement of AP.

In conclusion, PANoptosis plays an important role in the process of AP. Among them, inhibition of PANoptosis mediated by Zbp1-PANoptosome could improve the disease process of AP.

## Materials and methods

### Animals and reagents

C57BL/6 mice (SPF, 8 weeks old, 20 ± 2 g) were obtained from WeitongLihua Laboratory Animal Technology Company (Beijing, China). Animal experiments were approved by the Ethics Committee of Xuanwu Hospital, Capital Medical University, and conducted in accordance with the National Institutes of Health Guide for the Care and Use of Laboratory Animals. Mice were randomized into groups of 15 mice each (*n* = 15). Each mouse was individually weighed, and caerulein was injected intraperitoneally at a dose of 50 μg/kg every 1 h for 8 consecutive times. The control group was injected with normal saline. One hour after the last injection, mice were euthanized, and pancreatic tissue and serum were collected. Genetic intervention was achieved by intraperitoneal injection of siRNA. si-Zbp1 was injected 3 days prior to modeling at a dose of 3 nmol each.

Ceruletide was purchased from MCE (Nanjing, China). Anti-Nlrp3, anti-Caspase3, anti-Caspase1, anti-Gsdmd, anti-Pmlkl, anti-Ripk1, ani-Ripk3, and anti-Caspase8 antibodies were purchased from Abcam (Shanghai, China), while anti-Zbp1, anti-F4/80, anti-TNFα, anti-IL6, and anti-MPO antibodies were purchased from Proteintech (Wuhan, China). Anti-Bclxl was purchased from Abclonal (Wuhan, China). ELISA assay kits were purchased from SAB (Nanjing, China).

### Sequencing of the mouse pancreatic transcriptome

Pancreatic tissues were collected for RNA sequencing from mice in the Control group (*n* = 3) and AP group (*n* = 3). Fresh mouse pancreas tissues were used for RNA extraction as described in previous articles, and onboard assays were performed after passing quality control [36].

### Data acquisition and processing

The mouse pancreas dataset GSE65146 was obtained from the GEO database, and genes for apoptosis, necroptosis, and pyroptosis were retrieved from the literature. The mouse transcriptome data and the GSE65146 public database dataset were processed using R software. Differential gene analysis was performed using the “limma” package, and GSEA analysis was used to analyze the relationship between AP and PANoptosis. In addition, GO and KEGG enrichment were also performed using R. Protein interactions were analyzed using the STRING website and Cytoscape software.

### Biochemical and enzyme-linked immunosorbent assay (ELISA) assays

Mouse blood samples were collected, kept at room temperature for more than 4 h, and centrifuged at 1000×*g* for 15 min at 4 °C to obtain serum samples. Serum amylase and lipase were detected by assay kits (Nanjing Jiancheng). Elisa was used to measure serum TNFα.

### H&E staining

Fresh mouse pancreas was processed by fixation, embedding, sectioning, and staining with hematoxylin and eosin (H&E) to visualize the degree of inflammation and tissue damage in pancreatic tissues by light microscopy. We applied the scoring system defined by Kusshe et al. and the final scores of each group were summarized [37].

### Immunohistochemistry (IHC)

This is described in our previous article. Briefly, processed sections of mouse pancreas were incubated with antibodies (TNF-α, IL6, MPO, F4/80, Zbp1) overnight at 4 °C, followed by incubation with the corresponding secondary antibodies. The stained sections were visualized by light microscopy [36, 38].

### Immunofluorescence (IF)

The prepared tissues were blocked with sheep serum and incubated with primary antibodies as described above overnight and with the corresponding secondary antibodies for 1 h the next day. 4′,6-Diamidino-2-phenylindole (DAPI, Abcam) was used for nuclear staining. Stained sections were visualized by fluorescence microscopy.

### Cell culture and processing

The cell line 266-6 was purchased from ProCell (Danvers, MA). 266-6 cells were plated in DMEM culture medium containing 10% fetal bovine serum. The in vitro model was stimulated with 100 nM caerulein and 500 ng/ml trypsin for 24 h.

### Cell siRNA transfection

Cells were cultured in six-well plates, and transfection was performed when cells were in good condition. 1750 ml OPTI-MEM was added to the six-well plate, and then a mixture of si-Zbp1 and transfected liposomes was added to the transfection wells to make the final concentration of siRNA 100 nM. At 6 h after transfection, the culture medium was changed to DMEM containing 10% fetal bovine serum, and protein assay was performed at 48 h.

### Detection of apoptosis and necrosis

Cells were cultured in six-well plates and stimulated for modeling 24 h after transfection with siRNA. Cell death assay was performed 8–12 h after modeling. Cells were washed with precooled PBS, PE Annexin V and 7-AAD dye were added and incubated for 15 min in the dark before detection by flow cytometry.

### Western blot

As described in previous articles, treated cells were washed three times with PBS, lysate containing protease and phosphatase inhibitors was added, and proteins were scraped on ice [38–40]. The liquid was transferred to a 1.5 ml EP tube and kept on ice. It was shaken every 10 min for three consecutive times. Then the EP tube was centrifuged at 12,000 rpm at 4 °C for 10 min. The supernatant was collected and added to the sample buffer, and the metal bath was kept at 100 °C for 10 min. The concentration of the prepared proteins was determined by the BCA method, and then an equal amount of sample was taken in each well for gel electrophoresis. The primary antibody was incubated overnight at 4 °C. After TBST washing the next day, the corresponding secondary antibody was incubated at room temperature for 2 h. TBST was washed three times, and then exposure was performed. The results of the bands were analyzed using Image J.

### Statistical analysis

Data were expressed as mean ± standard deviation of at least three independent experiments and analyzed using GraphPad Prism. Comparison of two groups was analyzed by *t* test, and three or more groups were analyzed by one-way ANOVA. *P* < 0.05 was considered statistically significant (****P < 0.0001, ***P < 0.001, **P < 0.01, *P < 0.05, ns *P* > 0.05).

## Supplementary information

Western blot

## Data Availability

Data will be made available on request.
