# Supplementary material for: Inhibition of Zbp1-PANoptosome-mediated PANoptosis effectively attenuates acute pancreatitis
Source: Cell Death Discov. 2025 Apr 16;11:180. doi: 10.1038/s41420-025-02451-7 (PMC12003674; doi:10.1038/s41420-025-02451-7)
Supplement: Supplementary file 1 — Western blot [file 41420_2025_2451_MOESM1_ESM.docx]

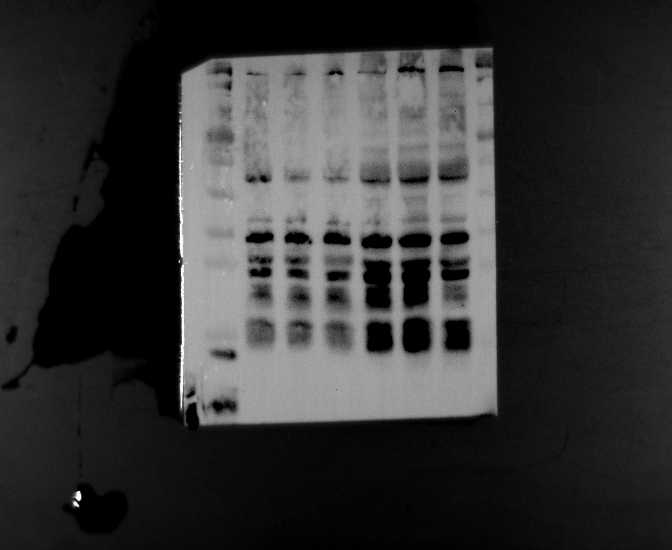

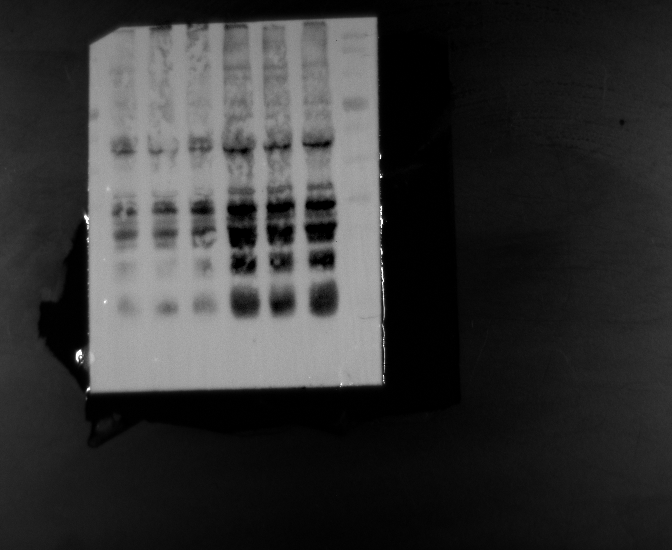


RIPK3

p-RIPK3


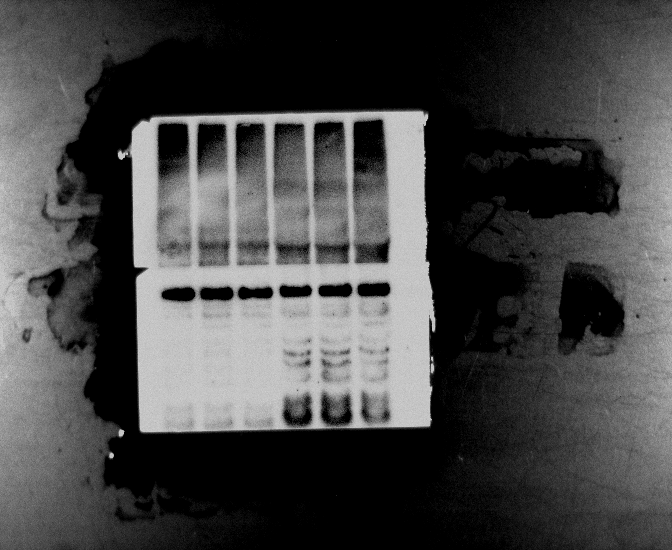

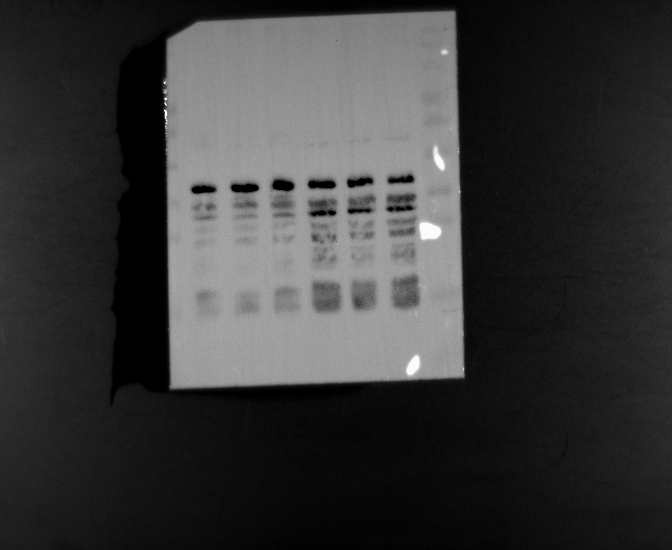


GAPDH

Gsdmd

GAPDH

MLKL

GAPDH


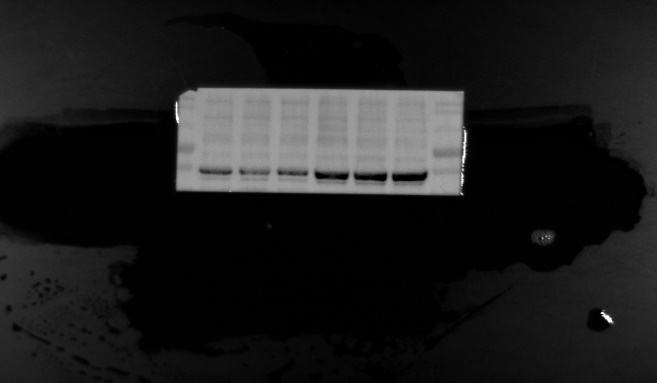

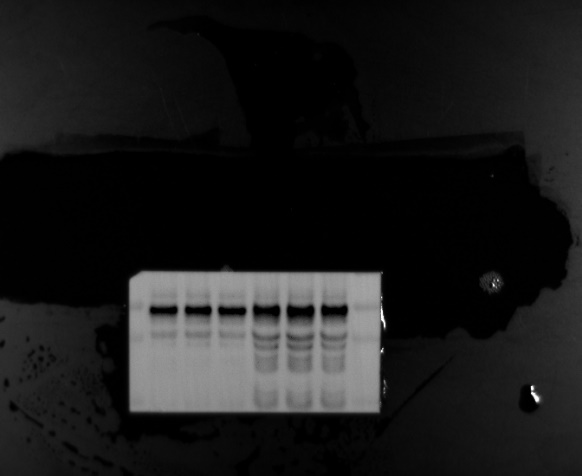


Cas3

Zbp1


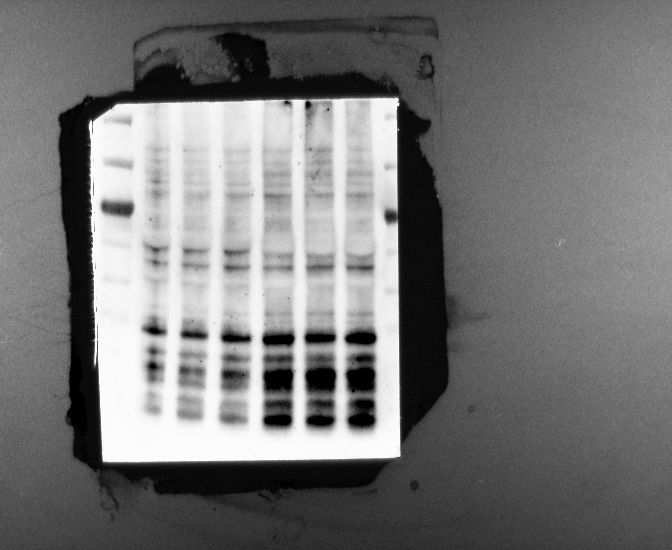


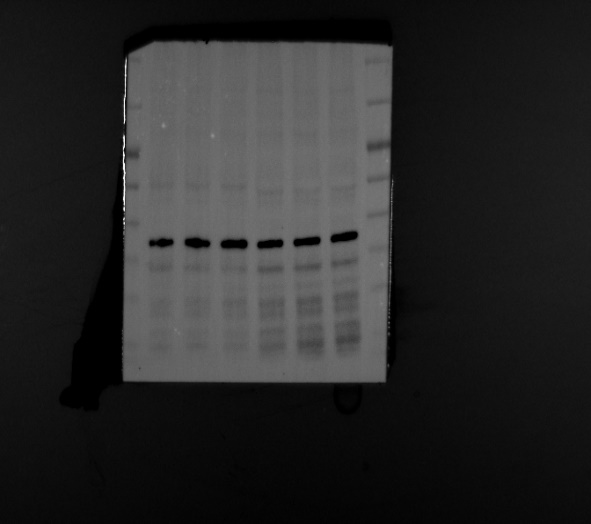


GAPDH

Gsdmd




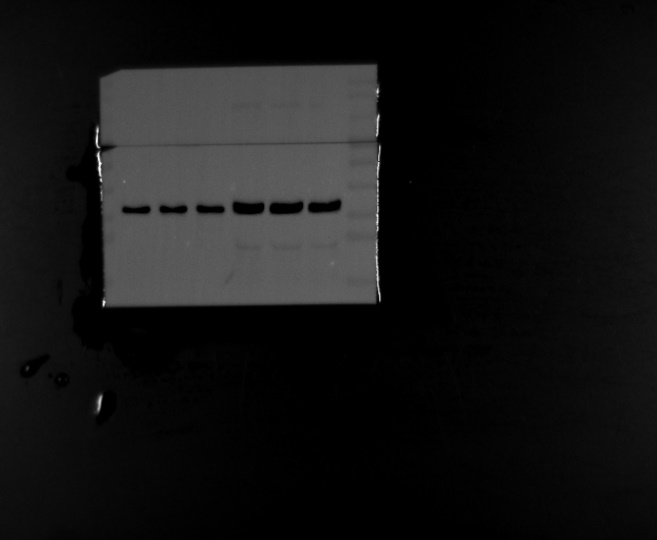


Nlrp3

GAPDH


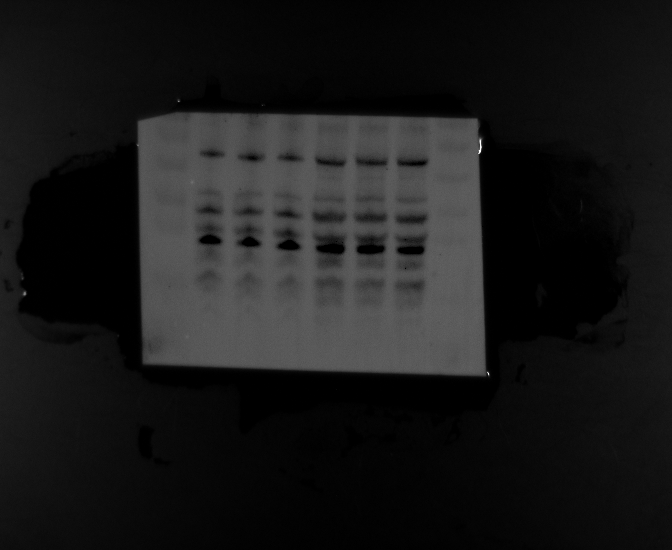

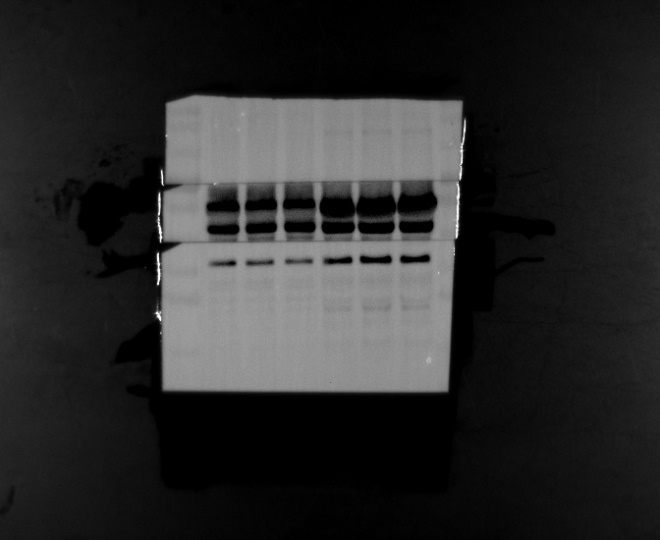


cas1

Gas8

GAPDH




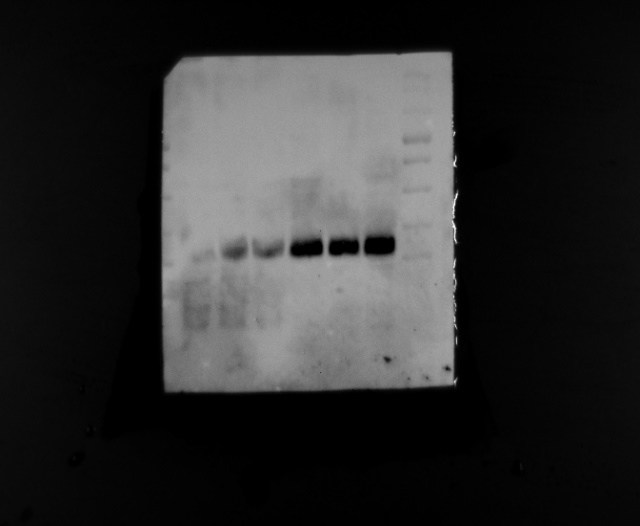


GAPDH

BCL-xl


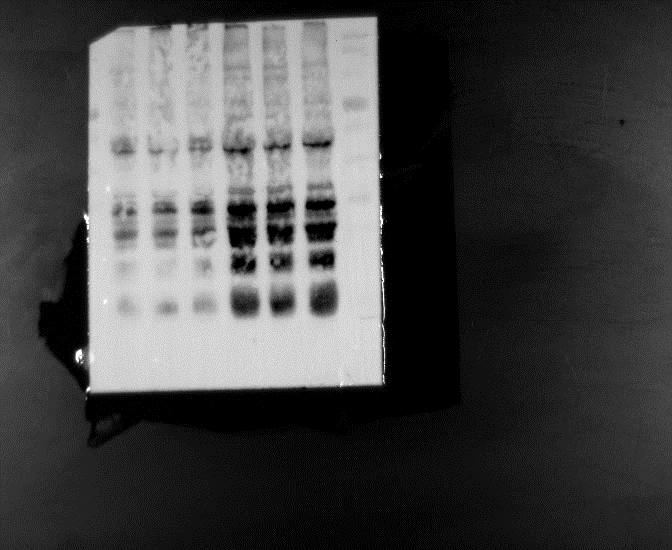


P-MLKL


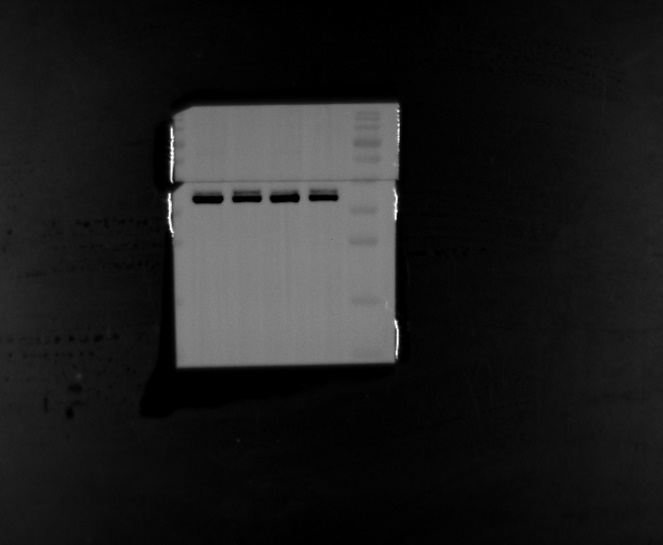

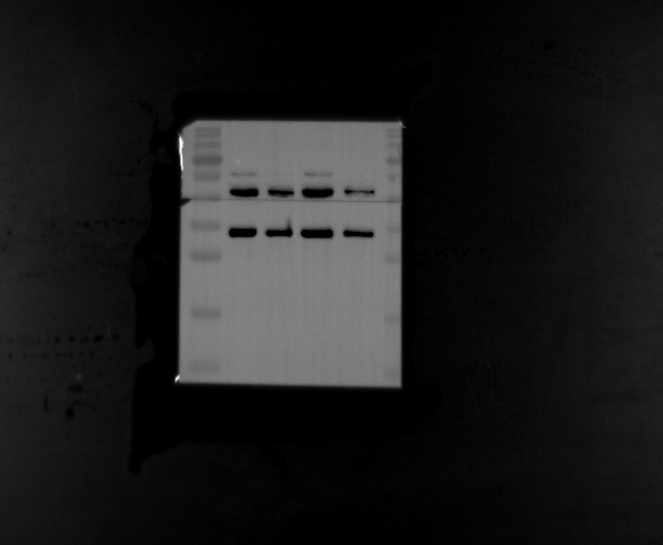


Gas3

Gas8

GAPDH


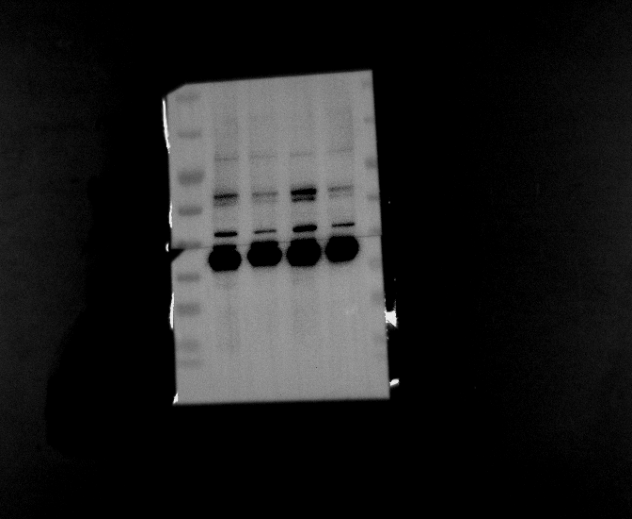

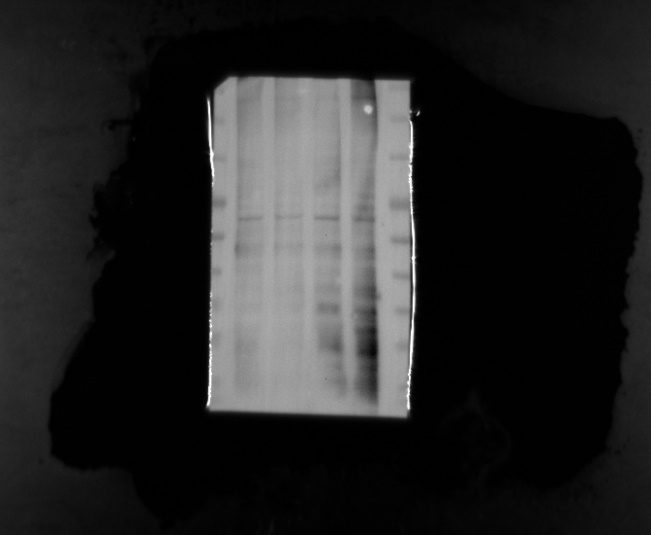


Zbp1

P-RIPK1

GAPDH


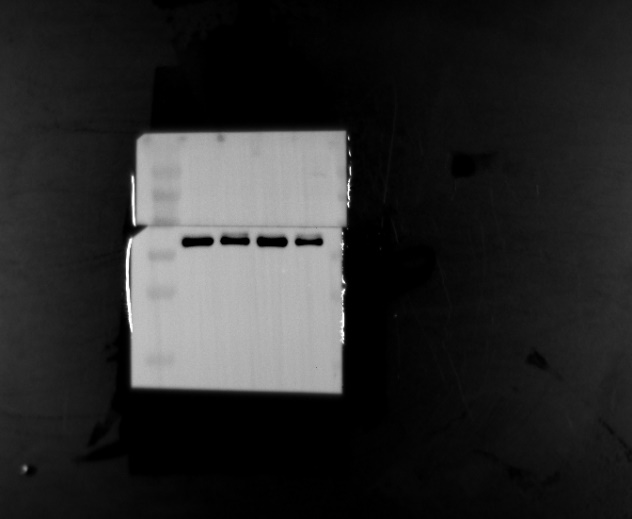

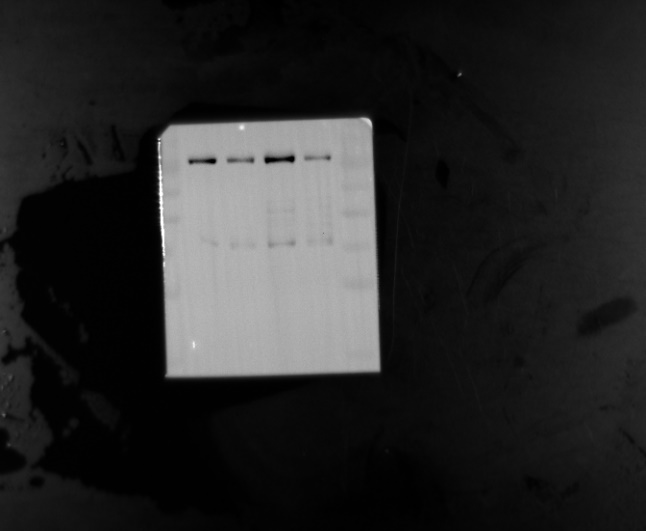


RIPK1

GAPDH




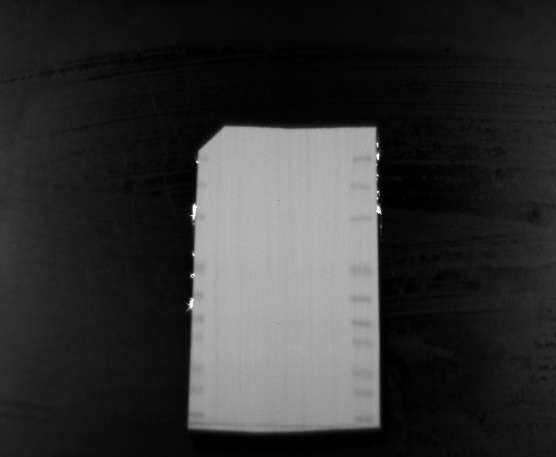


P-RIPK3


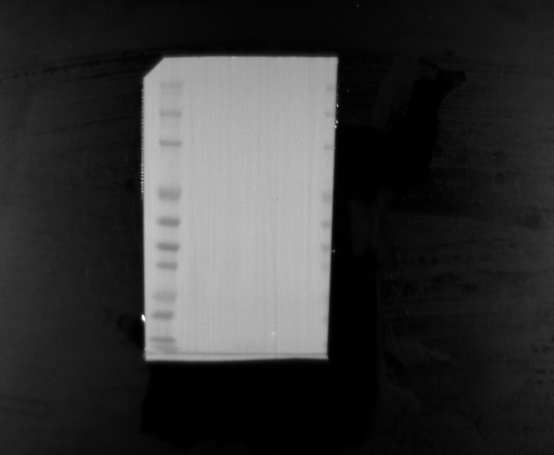

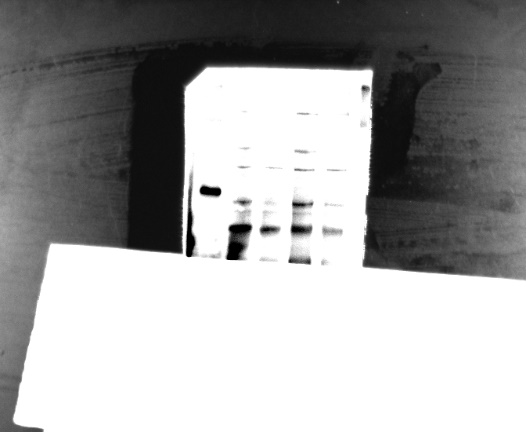


RIPK3


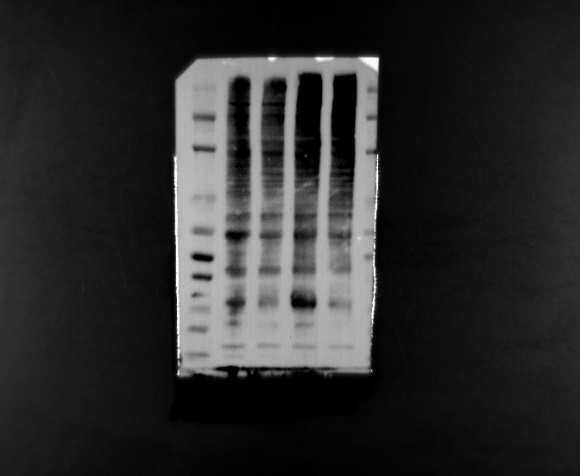

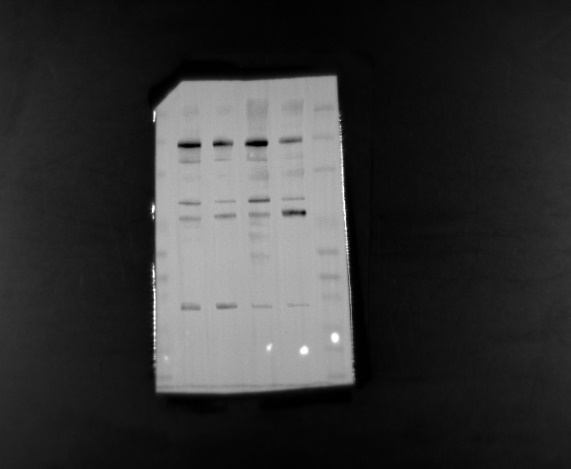


Nlrp3

P-RIPK1
